# Improving the Mechanical Response of Al–Mg–Si 6082 Structural Alloys during High-Temperature Exposure through Dispersoid Strengthening

**DOI:** 10.3390/ma13225295

**Published:** 2020-11-23

**Authors:** Jovid Rakhmonov, Kun Liu, Paul Rometsch, Nick Parson, X.-Grant Chen

**Affiliations:** 1 Department of Applied Science, University of Quebec at Chicoutimi, Saguenay, QC G7H 2B1, Canada; kun.liu@uqac.ca; 2 Arvida Research and Development Center, Rio Tinto Aluminum, Saguenay, QC G7S 4K8, Canada; Paul.Rometsch@riotinto.com (P.R.); nick.parson@riotinto.com (N.P.)

**Keywords:** Al–Mg–Si 6082 alloys, microstructure, high-temperature mechanical properties, residual mechanical behavior, α-Al(MnFe)Si dispersoids

## Abstract

The feasibility and efficacy of improving the mechanical response of Al–Mg–Si 6082 structural alloys during high temperature exposure through the incorporation of a high number of α-dispersoids in the aluminum matrix were investigated. The mechanical response of the alloys was characterized based on the instantaneous high-temperature and residual room-temperature strengths during and after isothermal exposure at various temperatures and durations. When exposed to 200 °C, the yield strength (YS) of the alloys was largely governed by β” precipitates. At 300 °C, β” transformed into coarse β’, thereby leading to the degradation of the instantaneous and residual YSs of the alloys. The strength improvement by the fine and dense dispersoids became evident owing to their complementary strengthening effect. At higher exposure temperatures (350–450 °C), the further improvement of the mechanical response became much more pronounced for the alloy containing fine and dense dispersoids. Its instantaneous YS was improved by 150–180% relative to the base alloy free of dispersoids, and the residual YS was raised by 140% after being exposed to 400–450 °C for 2 h. The results demonstrate that introducing thermally stable dispersoids is a cost-effective and promising approach for improving the mechanical response of aluminum structures during high temperature exposure.

## 1. Introduction

Owing to their preferable strength-to-weight ratio, good corrosion resistance, and weldability, Al–Mg–Si 6xxx alloys (typically 6061 and 6082 alloys) are increasingly used in load-bearing structural applications, such as land-based vehicles, marine crafts, light rails, bridge decks, off-shore platforms, and building structures [1,2,3,4,5,6]. Such aluminum structures can be subjected to unintentional fire exposures; therefore, fire safety is a major concern in their design and applications [3,4,7].

To evaluate the mechanical behavior of aluminum alloys under fire conditions, different test methods can be adopted [3], such as transient and steady-state mechanical tests, as well as the creep test. Based on data from steady-state tests, codes and regulations describing the design requirements for fire-prone aluminum structures have been established [8]. Owing to the lower melting temperature of aluminum alloys compared to those of common structural metals (e.g., iron and steel), the temperature range representative of realistic fire events and relevant for the mechanical properties is 150–450 °C [4]. In addition to the temperature, the exposure period at a given temperature is a key parameter that determines the resistance of aluminum structures to failure during and after fire. Most studies [7] and norms [8] indicate that the significant exposure time is between 0.5 and 2 h at a given temperature; 0.5 h refers to the critical time for safe evacuations and 2 h to the time the structure needs to maintain the adequate strength before the fire is extinguished [3,8]. However, in most studies, the constitutive behavior of aluminum structural materials at various temperatures and exposure periods have been assessed without considering the microstructural changes during the fire. Understanding the evolution of the microstructure and mechanical properties under fire conditions is essential for tailoring microstructures such that the aluminum structures perform better in fire events. Moreover, the number of studies of the high-temperature mechanical behavior of Al–Mg–Si alloys under various fire exposure severities is limited.

Al–Mg–Si 6xxx alloys are heat-treatable alloys, and their primary strengthening mechanism is the precipitation of nanoscale β”/β’-MgSi precipitates, which significantly increase the room temperature strength. However, these alloys experience a significant strength drop when exposed to temperatures higher than 200 °C, since the β”/β’ precipitates are highly sensitive to elevated temperature [9,10,11]. The damage extent of the properties of aluminum alloys depends on the fire exposure conditions (temperature and time). The coarsening and subsequent phase transformation of strengthening β”/β’ precipitates in 6xxx alloys are the main factors contributing to the degradation of the mechanical properties at elevated temperatures [1,3,12].

Recent studies have revealed that the mechanical properties (strength and creep resistance) of aluminum alloys at elevated temperatures can be substantially improved by introducing a high number of thermally stable α-Al(MnFe)Si dispersoids (referred as α-dispersoids hereafter) [13,14,15,16,17]. The most significant advantage of the dispersoid-strengthening over the traditional precipitation-strengthening in 6xxx alloys is their excellent thermal stability at elevated temperatures. The α-dispersoids can be formed in an α-Al matrix during the homogenization of Mn-containing 6082 alloys [18,19]. However, these dispersoids are coarse (diameter ranging from 100–200 nm) and exhibit low density, which leads to an insufficient strengthening effect [20]. In general, the efficiency of the dispersoid-strengthening effect depends highly on their size and density. The optimization of homogenization conditions to induce the precipitation of fine and dense dispersoids will be a new avenue to improve the high-temperature mechanical properties, which increase the time to failure/collapse of the aluminum 6082 structures, and hence improve the safety in case of a fire.

Furthermore, the post-fire (residual) mechanical behavior of aluminum structures is a key parameter that determines the stability and integrity of the entire structure [21]. After the fire, the aluminum structures (members) might be replaced, repaired, or directly reused, depending on the severity of the fire damage [2,21]. The residual mechanical behavior of aluminum alloys after a fire has been investigated in previous studies [2,21]. The residual yield strength (YS) of 6082-T6 alloy exposed to 450 °C and then cooled to room temperature has been reported to be 65 MPa, which was approximately 20% of the original YS before the high-temperature exposure [21]. The possibility of retaining fineness and high density of α-dispersoids in the microstructure of 6082 alloys during thermomechanical processes might also provide the aluminum structures with higher residual strength and better fire resistance, in light of the highly diminishing strengthening effect of β”/β’ precipitates upon thermal exposure.

The aims of this study were (1) to evaluate the microstructural evolution and associated mechanical properties of 6082 structural alloys exposed to different temperatures and times (simulated fire exposure), and (2) to assess the feasibility and efficacy of enhancing the mechanical response of 6082 structural alloys by the incorporation of fine and dense α-dispersoids, in addition to the β”-MgSi precipitates, into their microstructure.

## 2. Materials and Methods 

Two 6082 type alloys (without and with Mn) were prepared and cast in direct chill (DC)-cast billets with diameters of 101 mm. The chemical compositions of the alloys analyzed by optical emission spectroscopy are provided in Table 1. To promote the precipitation of a large amount of dispersoids [11], the DC-cast billets of the alloys were heat-treated at a relatively low temperature (400 °C) for 5 h before the extrusion process [11]; the 0.72Mn alloy treated under this condition is referred to as “0.72Mn(L)”. In addition, some DC-cast billets of the 0.72Mn alloy were heat-treated at 550 °C for 5 h, which are hereafter referred to as “0.72Mn(H)”. This typical industrial high temperature homogenization results in few and coarse dispersoids in the aluminum matrix [18,19]. Before the extrusion, all billets were inductively heated to 500 °C and then quickly transferred to the extrusion press. The extrusion was conducted at a ram speed of 10 mm/s to produce rods with diameters of 17.8 mm. Subsequently, the rods exiting the die traveled through a bath with agitating water, which served as a water quenching treatment. The rods were then subjected to an artificial aging at 180 °C for 5 h (T5 treatment).

To determine the mechanical behavior of the experimental alloys, compressive YS tests were conducted with a Gleeble 3800 thermomechanical simulator (Dynamic Systems Inc., Austin, TX, USA) unit at room and elevated temperatures. The mechanical properties of the three alloy variants to the thermal exposure were characterized by steady-state mechanical testing [3]. Different exposure temperatures (200, 300, 350, 400, and 450 °C) and typical two exposure times (0.5 h and 2 h) were chosen in the experiment. T5-treated rods from the three alloy variants were heated to the target exposure temperatures with a heating rate of 0.25 °C/s and held either for 0.5 h or 2 h, followed by water quenching. Cylindrical specimens of 15 mm height and 10 mm diameter were machined from the thermally exposed extrusion rods. During the Gleeble compression tests, the specimens were heated to the same target temperature at a rate of 2 °C/s, and held for 180 s to ensure a uniform temperature distribution. Subsequently, the specimens were compressed at a strain rate of 10^−3^ s^−1^ to reach a total strain of 0.2. Test reproducibility was ensured by testing at least three samples per condition. 

An optical microscope (OM, Nikon Instruments Inc., Tokyo, Japan), a scanning electron microscope (SEM, JEOL, Tokyo, Japan) equipped with electron backscatter diffraction (EBSD), and a transmission electron microscope (TEM, JEOL JEM-2100, Tokyo, Japan) were used to characterize the microstructure of the alloys. The grain structures after the extrusion and after thermal exposure were investigated by EBSD (samples were sectioned in the extrusion direction). The precipitates and dispersoids in the alloys under T5 conditions and after thermal exposure were characterized with the TEM, and TEM thin foils were obtained by electro-chemical polishing. The characteristics of the β” precipitates (e.g., average volume ($V_{p}$), number density ($N_{v}$), and volume fraction ($f_{p}$)) were quantified with the method in [22]. Moreover, the dispersoids were quantitatively characterized based on their average diameter ($\bar{D}$), number density ($N_{v}$), and volume fraction ($f_{d}$), which were determined according to the methodology provided in [23]. 

## 3. Results

### 3.1. Microstructure and Mechanical Properties under T5 Conditions

**Figure 1:**
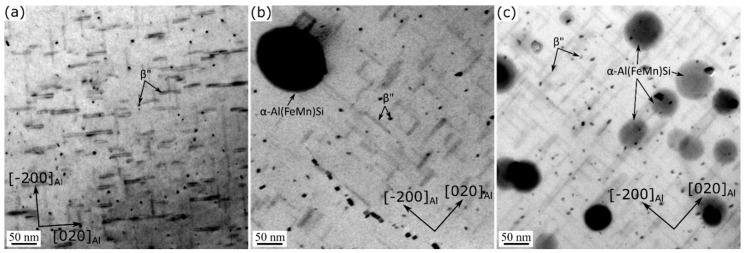
Bright-field TEM images of precipitates and dispersoids in aluminum matrices of T5-treated (**a**) 0Mn, (**b**) 0.72Mn(H), and (**c**) 0.72Mn(L) alloys.

Figure 1 shows the typical TEM images of the experimental alloys under T5 conditions. The results reveal uniformly distributed, fine, needle-shaped precipitates (approximately 4 nm diameter and 30 nm length) in the aluminum matrix of all three alloy variants. According to their morphology and size, the precipitates were identified as β” phase [24]. The quantitative results of the β” precipitates are listed in Table 2. Compared to the 0Mn base alloy, the Mn-containing alloys were characterized by higher number densities and volume fractions of the β” phase. 

**Figure 2:**
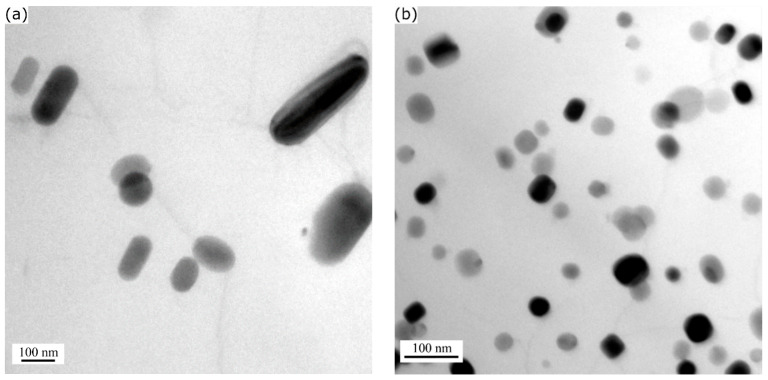
TEM images showing α-Al(FeMn)Si dispersoids embedded in aluminum matrices of (**a**) 0.72Mn(H) and (**b**) 0.72Mn(L) alloys. Images were taken near [001]_Al_ zone axes.

A key difference between the TEM microstructures of the various alloy conditions was the presence of a number of dispersoids in addition to the β” precipitates in the 0.72Mn(H) (Figure 1b) and 0.72Mn(L) (Figure 1c) alloys. The dispersoids in the 0.72Mn(L) alloy were much finer and denser than those in the 0.72Mn(H) alloy (see Figure 1b,c and Figure 2). To quantitatively compare the dispersoids, TEM investigations of the as-extruded Mn-containing alloys were conducted (Figure 2), because the aging treatment at 180 °C had no effect on the coarsening of the dispersoids. The dispersoids in the matrix were identified as α-Al(FeMn)Si [10,11], and their quantitative results are presented in Table 2. The average equivalent diameter ($\bar{D}$) and number density ($N_{v}$) of the dispersoids in the 0.72Mn(L) alloy were measured to be 40 nm and 430 µm^−3^, respectively (versus $\bar{D}$ of 146 nm and $N_{v}$ of 11 µm^−3^ in 0.72Mn(H) alloy), while the volume fractions of the dispersoids ($f_{d}$) in both alloys were similar (approximately 0.9%).

**Figure 3:**
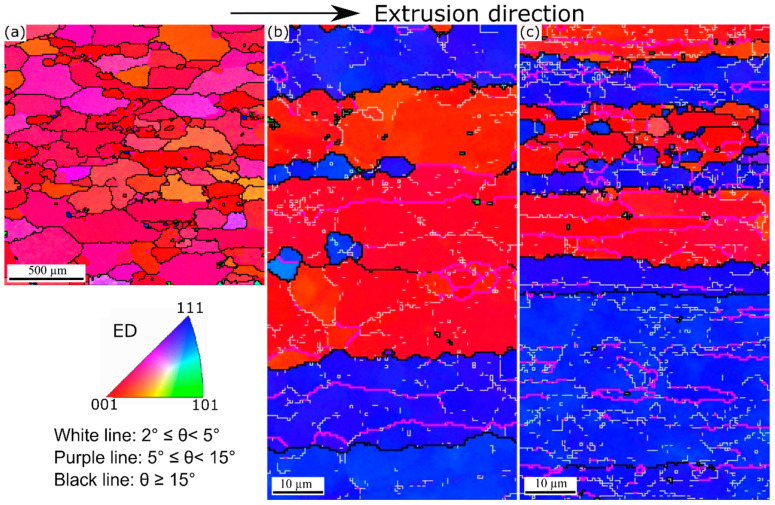
EBSD (inverse pole figure) maps representing extruded grain structures: (**a**) 0Mn, (**b**) 0.72Mn(H), and (**c**) 0.72Mn(L) alloys. Note that the EBSD scan area for 0Mn is much larger than for 0.72Mn(H) and 0.72Mn(L) alloys.

Based on the EBSD results of the as-extruded materials, it could be observed that the 0Mn alloy experienced full recrystallization during extrusion because the grains were coarse, equiaxed, and free of the substructure (Figure 3a) [25]. A <001> recrystallization texture highly dominated in this alloy (Figure 3a). The average equivalent diameter of the grains in 0Mn alloy were measured as ~220 µm. By contrast, the grain structures of the 0.72Mn(H) and 0.72Mn(L) alloys exhibited deformed and fibrous grains elongated along the extrusion direction (Figure 3b,c). The deformed grains appeared in one of the two <111> and <001> directions parallel to the extrusion axis, thereby indicating the evolution of <111> and <001> fiber textures during the axisymmetric deformation [26,27]. Evidently, only a dynamic recovery occurred in the 0.72Mn(H) and 0.72Mn(L) alloys during the extrusion, and the 0.72Mn(L) alloy exhibited a lower recovery level and a higher fraction of substructures (misorientation angle ranges between 5° and 15°) than did the 0.72Mn(H) alloy. 

**Figure 4:**
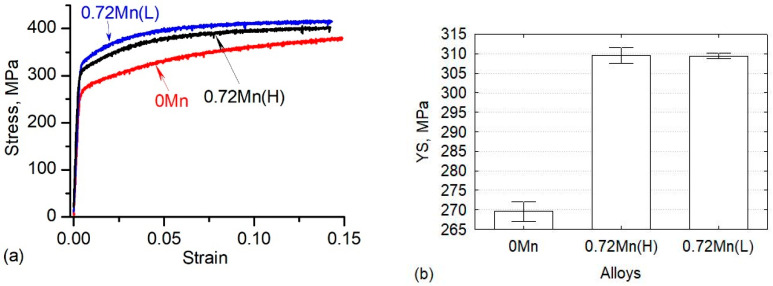
(**a**) Compressive true stress-strain curves and (**b**) compressive YS at room temperature of the alloys under T5 conditions.

Figure 4a displays the compressive true stress-strain curves at room temperature of the alloys in T5 state. Three alloys present similar work hardening behavior at the beginning of the compression test. With increasing the strain, the stress of the 0.72Mn(L) alloy was moderately higher than that of the 0.72Mn(H) alloy, but the stresses of both alloys were remarkably higher than that of the 0Mn base alloy. The obtained YSs (determined at 0.2% offset strain) of three alloys are shown in Figure 4b. It is evident that both Mn-containing alloys (0.72Mn(H) and 0.72Mn(L)) exhibited higher YS than the 0Mn base alloy (310 MPa versus 270 MPa, respectively). The YS of 6082 alloys under T5 conditions was mainly controlled by the nanoscale β” precipitates [22]. The higher strength of the Mn-containing alloys was primarily attributed to the higher number density and larger volume fraction of β” precipitates in the matrix (Table 2). Probably, the enhanced diffusion of solutes and accelerated nucleation and growth of β” precipitates in the deformed grains of both Mn-containing alloys led to the precipitation of a larger fraction of the β” phase than in the recrystallized 0Mn alloy during the subsequent aging treatment [9]. In addition, the higher strength of the Mn-containing alloys was partially contributed by their fibrous grain structure and texture compared to the fully recrystallized grains of the 0Mn alloy (Figure 3). Owing to the relatively large size and low density of dispersoids in the Mn-containing alloys, the strengthening effect of the dispersoids may have had a minor effect on the room temperature strength of the alloys relative to the predominant effect of β” precipitates. The fact that both 0.72Mn(L) and 0.72Mn(H) alloys under T5 condition exhibited similar YS indicated the limited strengthening effect of the dispersoids in the presence of a high number density of β” precipitates. 

### 3.2. Instantaneous High-Temperature Strength during Thermal Exposure

**Figure 5:**
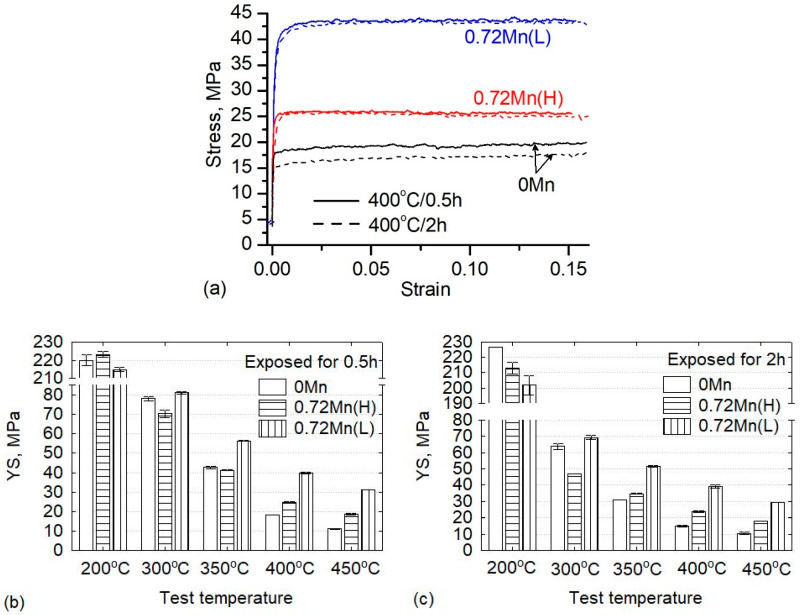
(**a**) Typical compressive true stress-strain curves at 400 °C and (**b**,**c**) instantaneous YS of three alloys at various temperatures, measured after being exposed to the test temperature for (**b**) 0.5 h and (**c**) 2 h.

The typical compressive true stress-strain curves at 400 °C are exemplarily displayed in Figure 5a. Obviously, the alloys show almost no work hardening behaviour at high temperature. Figure 5b,c shows the instantaneous YS of the three alloys at various temperatures and thermal exposures for 0.5 and 2 h. For both thermal exposure times, the strength of the alloys decreased with increasing exposure temperature. At 200 °C, all alloys thermally exposed for 0.5 h displayed a similar YS of approximately 220 MPa (Figure 5b). With increasing exposure time to 2 h, the YS of the 0Mn alloy increased slightly to 225 MPa, while the Mn-containing alloys displayed a slight decrease in their strength, and the 0.72Mn(L) alloy showed the lowest strength (202 MPa) (Figure 5c).

Compared to the strengths determined at 200 °C, the strengths of all three alloys at 300 °C decreased significantly. The YS at 300 °C and the thermal exposure of 0.5 h varied between 70 and 80 MPa. With increasing exposure time to 2 h, the alloys experienced a further reduction in their YSs. Interestingly, the 0.72Mn(L) alloy began to show its advantage in terms of strength, and its YS of 70 MPa for a 2 h exposure was higher than those of the other two alloys. The 0.72Mn(H) alloy exhibited the sharpest decrease in its YS (48 MPa). In addition, the YS of the 0Mn alloy reached 64 MPa. 

With further increasing temperature up to 450 °C, the strength of all alloys continued to decrease. The instantaneous YS of the 0Mn alloy decreased sharply at such high temperatures and attained only 11 MPa after an exposure at 450 °C; this represented only 5% of the YS after the exposure at 200 °C. The 0.72Mn(L) alloy showed the highest YS at these high temperatures; its YS values at each temperature for both exposure times were similar. Specifically, the YS values of the 0.72Mn(L) alloy after 2 h exposure reached 52, 40, and 31 MPa at 350, 400, and 450 °C, respectively, which were much higher than those of the 0.72Mn(H) and 0Mn alloys at the corresponding temperatures. For instance, after being exposed to 400–450 °C, the YS values of the 0.72Mn(L) alloy were 60%–75% and 150%–180% higher than those of the 0.72Mn(H) and 0Mn alloys, respectively. This demonstrates that the addition of Mn and the formation of thermally stable dispersoids had a great impact on the instantaneous YS at high temperatures (350–450 °C).

### 3.3. Residual Room-Temperature Strength after Thermal Exposure

**Figure 6:**
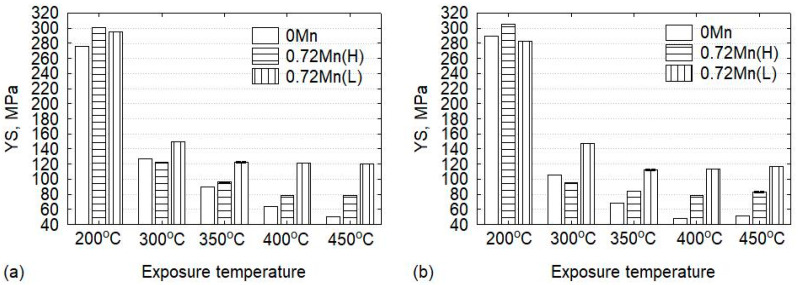
Residual room temperature YS of the experimental alloys; all alloys were pre-exposed to the test temperature for (**a**) 0.5 h and (**b**) 2 h.

The residual mechanical properties of the aluminum structure after the fire are a key parameter for evaluating the stability and integrity of the structure as well as the possibility of repair, reinforcement, and reuse [2,21]. To characterize the residual mechanical response, compressive YS tests were conducted on the alloys at room temperature after their exposure to elevated temperatures for two exposure times (0.5 and 2 h). The results are shown in Figure 6.

After the exposure at 200 °C, the three experimental alloys still exhibited high residual YSs, ranging between 280 and 300 MPa, which were at a similar level as the YSs under T5 conditions (Figure 4). Increasing the exposure time to 2 h exerted a negligible effect on the YSs of the alloys (Figure 6b). These results implied that the exposure to 200 °C for the investigated exposure times had no apparent deleterious effect on the mechanical properties of the aluminum structures. Therefore, they could be reused after fire.

Consistent with the high-temperature YS results, the exposure of the alloys to 300 °C resulted in a significant reduction in the residual YS (Figure 6). After thermal exposure for 2 h, the 0.72Mn(L) alloy reached a YS of 150 MPa, which largely exceeded those of the 0Mn and 0.72Mn(H) alloys (residual YSs of 105 and 95 MPa, respectively). It was evident that the residual YSs of the three alloys after the exposure to 300 °C were only 35%–45% of the original YSs under T5 conditions.

At higher exposure temperatures (350–450 °C), the residual YS of the 0.72Mn(L) alloy remained basically constant at the level of 115–120 MPa regardless of the exposure temperature and time. By contrast, the 0.72Mn(H) alloy exposed to 350 °C for 0.5 h exhibited a residual YS of 95 MPa. Furthermore, with increasing exposure temperature to 400–450 °C and exposure time to 2 h, the residual YS of this alloy decreased until it stabilized at 80 MPa. The residual mechanical response of the 0Mn alloy showed the worst performance. For instance, the 0Mn alloy exposed to 350 °C for 2 h attained a residual YS of 68.5 MPa; however, following the exposure to 400–450 °C, the residual YS decreased to 49 MPa, which represented only 18.5% of the original YS obtained prior to the high-temperature exposure. Hence, the 0.72Mn(L) alloy is a promising candidate for fire-susceptible aluminum structures, owing to its 50% and 140% higher residual YSs after exposures to 400–450 °C for 2 h than those of the 0.72Mn(H) and 0Mn alloys, respectively.

## 4. Discussion

Typically, Al–Mg–Si 6082 alloys experience predominant strengthening by the precipitation of nanoscale precipitates (particularly β”), which are formed during aging treatment [21]. Therefore, the precipitate stability during high temperature exposure determines the capacity of the aluminum structures to bear a certain amount of load, i.e., its strength during and after a fire [1]. With the addition of an adequate Mn to 6082 alloys and the appropriate heat treatment, a large number of thermally stable α-Al(FeMn)Si dispersoids can be formed during homogenization [11,20]; these contribute to preserving the high-temperature strength through dispersoid strengthening [10,11] and delayed restoration processes [25]. The extent of microstructural and mechanical stability of the aluminum alloys during high temperature exposure depends on the temperature and time [1].

At 200 °C, the overall instantaneous and residual YSs of all three alloys are still high and comparable, indicating that the β” precipitates formed during aging (T5 condition) control the strength of the alloys. In other words, the mechanical properties of the three alloys after exposures to 200 °C are not markedly deteriorated yet. The 0Mn alloy exhibits a slight increase in the instantaneous YS with prolonged thermal exposure, unlike the Mn-containing alloys (Figure 5). This can be explained by considering the precipitation structures under T5 conditions. Owing to the slower precipitation rate of the β” phase in the 0Mn alloy with recrystallized grain structure than that in the Mn-containing alloys with deformed grain structures, less dense and relatively finer β” precipitates were observed in the former alloy (Table 2). The prolonged exposure time for 2 h at 200 °C causes a further growth of the β” precipitates in the 0Mn alloy, thereby slightly increasing the YS relative to the value of the 0.5 h exposure. However, the Mn-containing alloys exhibit the over-aging phenomenon after a longer exposure. The higher amount of energy stored in the Mn-containing alloys (0.72Mn(L)) in the form of dislocations and subgrain boundaries (Figure 3) accelerates the coarsening of β” precipitates, thereby resulting in a moderate decrease in the strength and lower YS compared to that of the Mn-free 0Mn alloy. 

**Figure 7:**
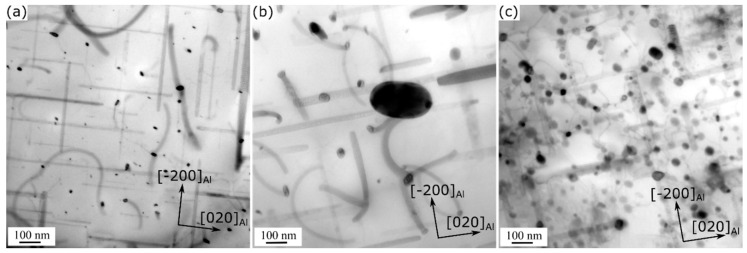
Bright-field TEM images of (**a**) 0Mn, (**b**) 0.72Mn(H), and (**c**) 0.72Mn(L) alloys exposed to 300 °C for 2 h after T5 treatment.

The three alloys exposed to 300 °C exhibit significant strength reductions, which indicates relevant changes in the strengthening precipitates. In fact, the TEM results of the alloys exposed to 300 °C for 2 h reveal the presence of coarse rod-shaped precipitates (Figure 7), which were identified as β’ phase based on their size and morphology [24]. The coarse β’ precipitates are known to be less effective in alloy strengthening [24,28,29]. Moreover, by comparing the characteristics of the β’ precipitates of the various alloys, it can be observed that the β’ precipitates in the 0Mn alloy are finer and denser than those in the Mn-containing alloys (Figure 7). The difference in the sizes and densities of the β’ precipitates between the 0Mn and Mn-containing alloys is likely associated with their initial as-extruded grain structures and, more specifically, with the higher rate of solute diffusion in the deformed grains of the Mn-containing alloys, which results in a quick coarsening of the β’ precipitates during thermal exposure [9]. This also explains why the 0Mn alloy possesses a higher YS than the 0.72Mn(H) alloy (for instance, 65 MPa versus 47 MPa after 2 h exposure at 300 °C, respectively). The β’ precipitates in the 0.72Mn(L) alloy are the same as those in the 0.72Mn(H) alloy. However, the 0.72Mn(L) alloy possesses a high number of fine α-dispersoids in the matrix (Figure 7c). It is evident that the dispersoids show no coarsening tendency during the thermal exposure and thus provide additional strength. Therefore, this alloy exhibits the highest instantaneous YS after a 2 h exposure (70 MPa; Figure 5). Moreover, the residual YS at 300 °C of this alloy is also substantially higher than those of the other two alloys (Figure 6) owing to the complementary strengthening effect of the dispersoids.

**Figure 8:**
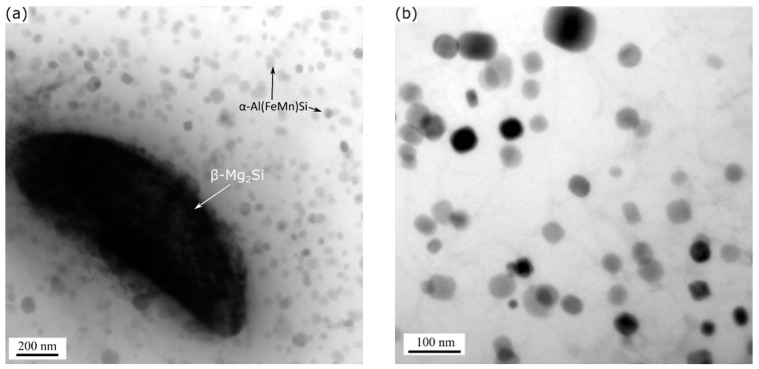
Bright-field TEM images showing (**a**) coarsened β-Mg_2_Si particles and (**b**) α-Al(FeMn)Si dispersoids in 0.72Mn(L) alloy exposed to 400 °C for 2 h after T5 treatment. Images were taken near [001]_Al_ zone axes.

At higher exposure temperatures (350–450 °C), the microstructures of the alloys keep changing with decreasing strength of the alloys. Figure 8 shows the typical TEM microstructure of the 0.72Mn(L) alloy exposed to 400 °C for 2 h. The Mg_2_Si precursor precipitates experience significant changes; they are transformed into μm-sized equilibrium β-Mg_2_Si particles (Figure 8a), while the α-dispersoids show no apparent coarsening (Figure 8b), because their dimensions are comparable to those in the as-extruded materials (Figure 2). The relatively high diffusion rates of Mg and Si in the aluminum matrix at such high temperatures cause the transformation of Mg_2_Si precursor precipitates into coarse equilibrium β particles, which lost their major strengthening effect [24]. In this case, the presence of thermally stable α-dispersoids contributes effectively to the strength of the Mn-containing alloys, becoming the dominant strengthening mechanism. Therefore, the dispersoid-free 0Mn alloy has very low instantaneous and residual YSs at elevated temperatures, while the 0.72Mn(L) alloy with fine and dense dispersoids has superior instantaneous and residual YSs, which results in the best mechanical response among the three alloys.

**Figure 9:**
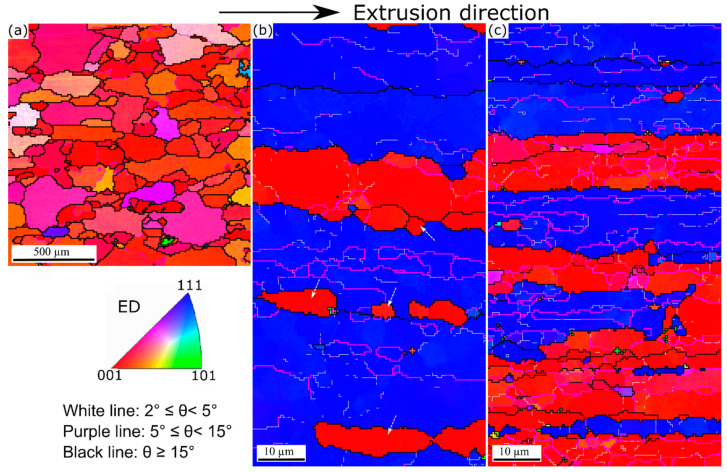
EBSD (inverse pole figure) maps of (**a**) 0Mn, (**b**) 0.72Mn(H), and (**c**) 0.72Mn(L) alloys, exposed to 400 °C for 2 h. Note that the EBSD scan area for 0Mn is much larger than for 0.72Mn(H) and 0.72Mn(L) alloys.

The grain structures of the three alloys after the high-temperature exposure (400 °C for 2 h) were investigated with EBSD (Figure 9). The 0Mn alloy maintains fully recrystallized grain structure (Figure 9a) because recrystallization has already occurred during extrusion (Figure 3a). The average equivalent diameter of grains in 0Mn alloy is ~220 µm, which is quite the same as that of the as-extruded 0Mn alloy (Figure 3a). This implies that no appreciable grain growth occurred during the high-temperature exposure. The 0.72Mn(H) alloy retains a large part of the recovered grain structure with some recrystallized grains on the grain boundaries (see arrows in Figure 9b). As previously mentioned, the 0.72Mn(H) alloy contains a number of α-dispersoids, which promote the recrystallization retardation owing to their pinning effect; nevertheless, it is less effective than that of the 0.72Mn(L) alloy. The fact that the 0.72Mn(H) alloy exhibits a higher residual YS than the 0Mn alloy at higher exposure temperatures (350–450 °C; Figure 6) can be partially attributed to their recovered grain structures, which enable better subgrain and strain hardening [9]. On the other hand, the 0.72Mn(L) alloy maintains its deformed and fibrous grain structure (Figure 9c) owing to the strong pinning effect of a large number of α-dispersoids, which inhibit recrystallization during the high-temperature exposure. The stable and high residual YSs at these high temperatures are contributed to a certain extent by the non-recrystallized grain structure owing to the subgrain strengthening effect.

In brief, at a low exposure temperature (200 °C), the fine nanoscale β” precipitates control the strength of Al–Mg–Si 6082 alloys, and the mechanical properties of aluminum structural alloys remain almost undeteriorated. With increasing exposure temperature to 300 °C, the β” precipitates transformed into coarse β’ precipitates, thereby resulting in a significant reduction in the alloy strength. Owing to a large number of fine and dense dispersoids, the 0.72Mn(L) alloy begins to show its superior mechanical response and exhibits remarkably higher instantaneous and residual YSs than the base 0Mn alloy and 0.72Mn(H) alloy, owing to the complementary strengthening effect of the thermally stable dispersoids. At higher exposure temperatures (350–450 °C), the Mg_2_Si precursor precipitates transform into coarse equilibrium β-Mg_2_Si particles and lose their strengthening effect. Moreover, the strength of the base 0Mn alloy decreases with increasing exposure temperature, and the alloy possesses very low instantaneous and residual YSs at high temperatures. On the other hand, the dispersoids show no apparent coarsening and effectively contribute to alloy strength. Therefore, the 0.72Mn(L) alloy with fine and dense dispersoids exhibits the best mechanical response at 350–450 °C among the three alloys. Hence, introducing thermally stable dispersoids by appropriate Mn alloying and heat treatments can be a cost-effective and promising approach for improving the mechanical response of aluminum structural alloys during high temperature exposure.

## 5. Conclusions

- The addition of Mn in a typical high-temperature homogenization treatment produced a number of α-Al(FeMn)Si dispersoids in the 0.72Mn(H) alloy, which improved the mechanical response during thermal exposure at 350–450 °C relative to that of the Mn-free base alloy. Moreover, the low-temperature homogenization treatment resulted in a high density of fine dispersoids in the 0.72Mn(L) alloy, which further improved the mechanical response substantially.
- At a low exposure temperature (200 °C), the instantaneous and residual YSs of the alloys were mainly governed by the β” precipitates, and the mechanical properties of the aluminum structural alloys were not markedly affected compared to their original strength under T5 condition.
- At a high exposure temperature of 300 °C, the β” precipitates transformed into coarse β’ precipitates, thereby resulting in a significant reduction in the alloy strength. The strength improvement due to the presence of fine and dense dispersoids became quite evident, because the instantaneous and residual strengths of the corresponding alloy (0.72Mn(L)) were higher than those of the alloys without dispersoids (0Mn) or with coarse dispersoids (0.72Mn(H)).
- At higher exposure temperatures (350–450 °C), the Mg_2_Si precursor precipitates transformed into coarse equilibrium β-Mg_2_Si particles and lost their strengthening effect, while the dispersoids resisted the coarsening and became the dominant strengthening contributor. The 0.72Mn(L) alloy containing fine and dense dispersoids displayed far superior instantaneous and residual YSs compared to the other two alloy variants and therefore the best mechanical response during high temperature exposure.
- The presence of thermally stable dispersoids effectively retarded the recrystallization during high temperature exposure, which improved the high-temperature mechanical properties to a certain extent.

## Figures and Tables

**Table 1:** Chemical compositions of experimental alloys (wt.%).

Alloys	Mg	Si	Mn	Fe	Ti	Al
0Mn (base)	0.83	1.01	0	0.22	0.018	Bal.
0.72Mn	0.84	1.02	0.72	0.23	0.016	Bal.

**Table 2:** Quantitative TEM results of β” precipitates and α-Al(FeMn)Si dispersoids in three alloys.

Alloy	β” Phase			α-Al(FeMn)Si Dispersoids		
	$V_{p},\;{\mathrm{nm}}^{3}$	$N_{v},\;{\mathrm{nm}}^{-3}$	$f_{p},\;\%$	$\bar{D}$	$N_{v},\;µm^{-3}$	$f_{d},\%$
0Mn	410	1.26 × 10^−5^	0.52	-	-	-
0.72Mn(H)	459	1.40 × 10^−5^	0.62	146	11	0.91
0.72Mn(L)	468	1.33 × 10^−5^	0.64	40	430	0.88
